# The high affinity selectin glycan ligand C2-O-sLe^x ^and mRNA transcripts of the core 2 β-1,6-*N*-acetylglusaminyltransferase (C2GnT1) gene are highly expressed in human colorectal adenocarcinomas

**DOI:** 10.1186/1471-2407-9-79

**Published:** 2009-03-06

**Authors:** Catherine A St Hill, Mariya Farooqui, Gregory Mitcheltree, H Evin Gulbahce, Jose Jessurun, Qing Cao, Bruce Walcheck

**Affiliations:** 1 Department of Veterinary Clinical Sciences, University of Minnesota, Room C339, Veterinary Medical Center, 1352 Boyd Avenue, St. Paul, MN 55108, USA; 2 Department of Laboratory Medicine and Pathology, University of Minnesota, 420 Delaware St SE, Minneapolis, MN 55455, USA; 3 Biostatistics and Informatics Shared Resource, University of Minnesota, 420 Delaware, Minneapolis, MN 55455, USA; 4 Department of Veterinary and Biomedical Sciences, University of Minnesota, 1988 Fitch Avenue, St Paul, MN 55108, USA

## Abstract

**Background:**

The metastasis of cancer cells and leukocyte extravasation into inflamed tissues share common features. Specialized carbohydrates modified with sialyl Lewis x (sLe^x^) antigens on leukocyte membranes are ligands for selectin adhesion molecules on activated vascular endothelial cells at inflammatory sites. The activity of the enzyme core 2 β1,6 *N*-acetylglucosaminyltransferase (C2GnT1) in leukocytes greatly increases their ability to bind to endothelial selectins. C2GnT1 is essential for the synthesis of core 2-branched O-linked carbohydrates terminated with sLe^x ^(C2-O-sLe^x^). Our goal was to determine the expression profiles of C2-O-sLe^x ^in the malignant progression and metastasis of colorectal adenocarcinomas. The well characterized CHO-131 monoclonal antibody (mAb) specifically recognizes C2-O-sLe^x ^present in human leukocytes and carcinoma cells. Using CHO-131 mAb, we investigated whether C2-O-sLe^x ^was present in 113 human primary colorectal adenocarcinomas, 10 colorectal adenomas, 46 metastatic liver tumors, 28 normal colorectal tissues, and 5 normal liver tissues by immunohistochemistry. We also examined mRNA levels of the enzyme core 2 β1,6-*N*-acetylglucosaminyltransferase (C2GnT1) in 20 well, 15 moderately, and 2 poorly differentiated colorectal adenocarcinomas, and in 5 normal colorectal tissues by using quantitative real-time polymerase chain reactions (RT-PCR).

**Results:**

We observed high reactivity with CHO-131 mAb in approximately 70% of colorectal carcinomas and 87% of metastatic liver tumors but a lack of reactivity in colorectal adenomas and normal colonic and liver tissues. Positive reactivity with CHO-131 mAb was very prominent in neoplastic colorectal glands of well to moderately differentiated adenocarcinomas. The most intense staining with CHO-131 mAb was observed at the advancing edge of tumors with the deepest invasive components.

Finally, we analyzed C2GnT1 mRNA levels in 37 colorectal adenocarcinomas and 5 normal colorectal tissues by RT-PCR. Significantly, we observed a greater than 15-fold increase in C2GnT1 mRNA levels in colorectal adenocarcinomas compared to normal colorectal tissues.

**Conclusion:**

C2-O-sLe^x^, detected by the CHO-131 mAb, is a tumor associated antigen whose expression is highly upregulated in colorectal adenocarcinomas and metastatic liver tumors compared to normal tissues. C2-O-sLe^x ^is a potentially useful early predictor of metastasis.

## Background

The malignant transformation of cells is associated with abnormal glycosylation, which results in the altered expression of carbohydrates on the surface of cancer cells [1]. Aberrant glycosylation profoundly impacts most, if not all processes involved in tumor cell invasion and metastasis. The glycosylation status of specific carbohydrate epitopes can modulate diverse cellular functions such as cell growth, adhesion, signal transduction, and motility. Human carcinomas express high levels of the sialyl Lewis x (sLe^x^) tetrasaccharide, a sialylated and fucosylated carbohydrate antigen, and its isomer sialyl Le^a^, which are associated with a greatly increased metastatic potential and a poor prognosis [2-7]. In particular, carbohydrate antigens such as sLe^x ^are thought to contribute to the metastatic process because sLe^x ^levels increase as colorectal adenocarcinomas progress from non-metastatic to metastatic tumors [3,8]. The status of sLe^x^, but not sLe^a^, in colorectal cancers was shown to be an independent predictive factor for disease recurrence, depth of tumor invasion, and histologic type [9,10].

**Figure 1:**
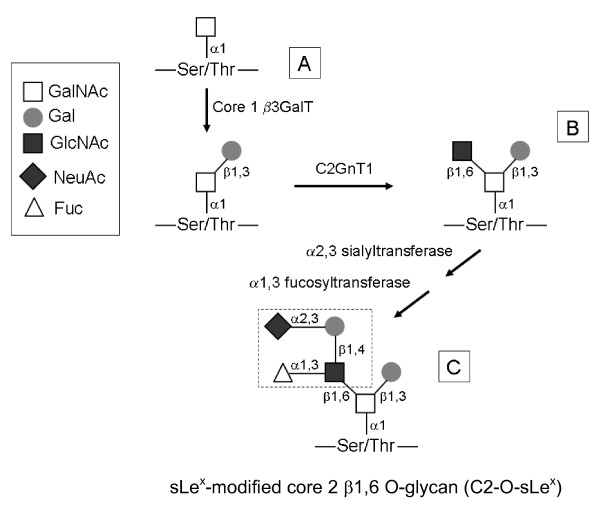
**Diagram of core 2 β1,6 O-glycan synthesis**. (A) Core 1 O-glycans are synthesized by addition of β1,3 galactose to N-acetylgalactosamine. (B) The C2GnT1 enzyme converts an unsubstituted core 1 O-glycan to a core 2 β1,6 O-glycan. (C) Core 2 can be further modified by α2,3 sialyltransferase and α1,3 fucosyltransferase, forming a sLe^x ^terminus (dotted box). These modifications result in the synthesis of the sLe^x^-modified core 2 β1,6 O-glycan (C2-O-sLe^x^) structure. The figure is simplified and some enzymatic steps are omitted for clarity. GalNAc, N-acetylgalactosamine; Gal, galactose; GlcNAc, N-acetylglucosamine; NeuAc, sialic acid; Fuc, fucose.

The sLe^x ^carbohydrate antigen can act as a ligand for the selectin family of adhesion molecules [11,12]. Studies using transfected cell lines [13-15] and knock out mice [16,17] revealed that selectin binding to sLe^x ^is greatly increased upon expression of the enzyme core 2 β1,6-*N*-acetylglucosaminyltransferase (C2GnT1). C2GnT1 catalyzes the synthesis of core 2 β1,6 branched O-glycans (C2-O-sLe^x^). O-glycosylation is initiated by tissue-specific addition of the *N*-acetylgalactosamine (GalNAc) residue to a serine or threonine of a protein. In subsequent enzymatic steps, the enzyme C2GnT1 adds a β1,6 branched linkage of GlcNAc to a core 1 O-glycan scaffold to form the core 2 β1,6 branched structure. The core 2 branch can be further extended by lactosamine units and eventually terminated by sialylation (α2,3-sialyltransferase) and fucosylation [α-(1,3)-fucosyltransferase] to form the sLe^x ^epitope, ultimately resulting in the synthesis of C2-O-sLe^x ^[18] (Figure 1).

C2-O-sLe^x ^decorates the human leukocyte mucin P-selectin glycoprotein ligand-1 (PSGL1, CD162) and confers high affinity binding of PSGL-1 to the selectins [19,20], a mechanism that is crucial for leukocyte migration during inflammatory events. Much stronger bonds are formed when leukocytes expressing cell surface C2-O-sLe^x ^bind to selectins than when sLe^x ^is present on other glycans. Evidence suggests that similar adhesion events may also occur in the dissemination of tumor cells via the circulatory system [21-24]. *In vitro *and *in vivo *studies have demonstrated that carbohydrate determinants, such as sLe^x^, on carcinoma cells are glycan ligands for the selectins [25-29]. Interactions of L-selectin on leukocytes [30], E- and P-selectin on the vascular endothelium [31-33], and P-selectin on platelets [34] with their ligands have been implicated to facilitate the metastasis of carcinoma cells. Thus far, however, the importance of C2-O-sLe^X ^in the progression and metastasis of colorectal carcinomas has not been directly examined. As with leukocytes, the expression of C2-O-sLe^x ^on carcinoma cells may confer greatly enhanced adhesion to the selectins and promote adherence, extravasation, and metastasis.

Previous studies have reported that C2GnT1 transcripts, and in some instances sLe^x^, were expressed in carcinomas and correlated with vessel invasion, depth of tumor invasion, and metastasis [3,35-37]. These studies provided indirect evidence that C2-O-sLe^x ^may participate in carcinoma progression but this relationship needs to be confirmed by the direct detection of C2-O-sLe^x ^on carcinoma cells. Detection of cell surface C2-O-sLe^x ^directly demonstrates the functional activity of C2GnT1. This distinction is important because other enzymes can compete with C2GnT1 and prevent the formation of C2-O-sLe^x ^in carcinoma cells [38]. Inhibition of C2GnT1 by other enzymes can result in the distribution of sLe^x ^on other carbohydrates, including O-glycans, N-glycans and glycolipids, that may have much lower affinities for selecting binding [39].

Recently, we have extensively characterized the monoclonal antibody (mAb) CHO-131 that specifically recognizes C2-O-sLe^x ^[40]. The binding specificity of CHO-131 mAb was evaluated using synthetic glycopeptides modeled after the N-terminus of human PSGL-1 containing precise O-glycan structures [40]. The reactivity of CHO-131 mAb, unlike that of anti-sLe^x ^antibodies, requires the functional activity of the glycosyltransferases C2GnT1, α2,3-sialyltransferase, and α1,3-fucosyltransferase and therefore was ideal for addressing our hypothesis [40-42]. CHO-131 mAb is not a function blocking antibody, however, and at high concentrations does not inhibit interactions of leukocyte C2-O-sLe^x ^on PSGL1 with P-selectin [40]. We have previously demonstrated that C2-O-sLe^x ^was expressed on a colorectal adenocarcinoma cell line that avidly attached to E-selectin [43]. The purpose of this study was to investigate the expression of C2-O-sLe^x ^in malignant and benign human colorectal tissues and hepatic metastases. We hypothesized that C2-O-sLe^x^, a high affinity selectin ligand, is abundantly expressed on carcinoma cells and is associated with malignant progression and metastasis of colorectal adenocarcinomas. In our approach, we used RT-PCR and immunohistochemistry to assess the levels of C2GnT1 mRNA and expression of C2-O-sLe^x ^in colorectal adenocarcinomas, adenomas, metastatic liver tumors and normal colorectal and liver tissues. We found higher expression of C2-O-sLe^x ^and correspondingly high C2GnT1 mRNA levels on colorectal adenocarcinomas compared to normal tissues. We also observed high expression of C2-O-sLe^x ^on liver metastases of colorectal adenocarcinomas. Based on these results, we propose that C2-O-sLe^x ^is a tumor associated antigen and a potential predictor of tumor progression and metastasis.

## Methods

### Tissue Samples

All tissues were used in accordance with the University of Minnesota's Institutional Review Board regulations. For the immunohistochemical staining procedures tissue samples were from males and females between the ages of 26 and 82 years. The tissue samples used for the RT-PCR analysis were from males and females between the ages of 31 and 93 years.

### Antibodies

CHO-131 mAb is a mouse IgM that binds the glycan structure C2-O-sLe^x ^and specifically recognizes the presence of this glycan in human leukocytes and carcinoma cells [40,43]. Mouse anti-CEA clone Col-1 mAb is a mouse IgG_2a _(Invitrogen, Carlsbad, CA) that specifically recognizes human carcinoembryonic antigen (CEA) in the cytoplasm and membrane of colorectal adenocarcinoma cells but does not react with normal colorectal tissues or with non-specific cross-reacting antigen. Pre-malignant cells, such as colorectal adenomas, also positively react with anti-CEA mAb. Mouse mAb Ab-3, clone Lu-5 is a mouse IgG_1 _(Thermo Fisher Scientific, Fremont, CA) and is a broad spectrum anti pan-keratin antibody which reacts with epithelial cells and differentiates epithelial from non-epithelial tumors. CSLEX 1 mAb, obtained from Dr. Bruce Walcheck, is a mouse IgM that recognizes the sLe^x ^epitope on the cell membrane.

The secondary antibodies used for CSLEX1 and CHO-131 mAb staining were mu-chain specific horseradish peroxidase (HRP) conjugated goat anti-mouse IgM (Invitrogen, Carlsbad, CA) and mu-chain specific alkaline phosphatase (AP) conjugated goat anti-mouse IgM (Chemicon International Inc., Billerica, MA) respectively. For Lu-5 and CEA staining, gamma-chain specific horseradish peroxidase conjugated goat anti-mouse IgG (Chemicon International Inc., Billerica, MA) was used as a secondary antibody. Mouse IgM, mouse IgG_2a _and mouse IgG_1 _(Invitrogen) were used as negative control antibodies for CHO-131, CEA, and Lu-5 mAb staining respectively at the same concentrations as the corresponding primary antibodies.

### Histopathology

Tissue sections used for immunohistochemical staining were 4-μm thickness, fixed in 10% neutral buffered formalin, and embedded in paraffin. We stained representative sections from each case with hematoxylin and eosin (H & E) according to standard procedures to evaluate tissue morphology. We examined 113 cases of primary colorectal adenocarcinomas, 46 cases of liver metastasis of colorectal adenocarcinomas, 10 cases of colorectal adenomas, 28 normal colorectal tissues, and 5 normal liver samples from pooled specimens that were obtained commercially from MaxArray colorectal adenocarcinoma/normal tissue microarrays (Invitrogen, Carlsbad, CA); AccuMax colon cancer and liver metastasis of colon cancer arrays (ISU ABXIS Co., Ltd, Seoul, South Korea); and from archived tissues (Tissue Procurement Facility, University of Minnesota, Minneapolis, MN). Serial sections were prepared from tissues stained with CHO-131 mAb of 113 colorectal adenocarcinomas, 38 metastatic liver tumors, 5 sections of normal colonic mucosa, and 5 sections of normal liver, and were stained with CSLEX1 mAb. An additional 21 primary colorectal adenocarcinomas (134 total adenocarcinomas) were stained with CSLEX1 mAb to verify localization of the sLe^x ^epitope.

A TissueScan colon cancer tissue qPCR array panel (Origene Technologies, Rockville, MD) was obtained commercially and used for evaluation of mRNA expression of C2GNT1 in colorectal tissues by RT-PCR. The panel consisted of cDNAs from tissue samples of 42 human colorectal adenocarcinomas and 6 normal colorectal tissues that were PCR-ready and normalized with β-actin by the company.

Tissues used in the immunohistochemical staining procedures and those from which mRNA was isolated for the quantitative real time-PCR (RT-PCR) experiments were classified as grades of well differentiated, moderately differentiated, and poorly differentiated that represented the differentiation status of tumor cells.

### Immunohistochemistry

Tumor sections for all staining procedures were deparaffinized in three changes of xylene, hydrated through 100-70% graded concentrations of ethanol and washed in distilled water and 1× Tris/Tween buffer, pH 7.6. To eliminate non-specific staining, tissue sections were incubated with protein block (ScyTek Laboratories, Logan, UT) for 10 minutes, excess block was removed and primary antibody was immediately applied to sections. Incubations with protein block and primary and secondary antibodies were carried out in a humidified chamber at 27 – 30°C. The remaining staining procedures were performed at room temperature. As negative controls, additional sections from each specimen were prepared by replacing the primary antibody with the corresponding isotype-matched, negative control antibody (Invitrogen, Carlsbad, CA) using similar conditions and antibody concentrations to determine the specificity of antibody binding. The primary antibody was removed by washing in 1× Tris/Tween buffer and secondary antibody was applied to tissue sections. Sections were washed, incubated with the chromagen, and counterstained with Mayer's hematoxylin (Dako, Carpinteria, CA). Sections were washed, dehydrated through 70 – 100% graded alcohols, 100% xylenes, and mounted. Stained sections were evaluated by light microscopy.

To detect C2-O-sLe^x^, tissue sections were stained with CHO-131 mAb at a concentration of 15 μg/ml. It was not necessary to perform antigen retrieval or blocking of endogenous peroxidase activity. CHO-131 mAb was applied to tissue sections for 2 hours. Sections were washed and incubated with mu-chain specific goat anti-mouse IgM-AP secondary antibody (16 μg/ml) for 30 minutes. After washing, tissue sections were incubated with a Vector Red alkaline phosphatase chromagen (Vector Laboratories, Burlingame, CA) for 2 minutes.

For detection of the sLe^x ^epitope, antigen retrieval was not performed. Endogenous peroxidase activity was blocked by incubation of sections with 3% hydrogen peroxide in 1× PBS for 15 minutes at room temperature. Tissue sections were stained with CSLEX1 mAb (15 μg/ml) for 2 hours, washed, and incubated with mμ-chain specific goat anti-mouse IgM-HRP (30 μg/ml) antibody for 30 minutes. Sections were washed and 3,3- diaminobenzidinetetrahydrochloride (DAB) chromagen (Vector Laboratories, Burlingame, CA) was applied for 5 minutes.

The HL-60 promyelocytic leukemia cell line (American Type Culture Collection, Manassas, VA) expresses high levels of membrane bound sLe^x^, high C2GnT1 activity [44], and in our hands expressed high levels of C2-O-sLe^x^. Cytospins of HL-60 cells (3 × 10^4 ^cells/sample) were generated using a Shandon-Southern SCA-0031 cytocentrifuge (Sewickley, PA, USA) at a speed of 1000 rpm for 5 minutes and were stained with CSLEX1 and CHO-131 mAbs as positive controls.

As additional positive controls, mouse anti-CEA mAb was used at a concentration of 1.6 μg/ml. Lu-5 mAb was used at a concentration of 0.5 μg/ml. The anti-CEA and Lu-5 mAb staining techniques were performed similarly to that for CSLEX1 mAb with some modifications. Proteinase K (Dako, Carpinteria, CA) was used for antigen retrieval in the Lu-5 but not in the CEA mAb staining procedures according to the manufacturer's instructions. Endogenous peroxidase activity was blocked in both Lu-5 and CEA mAb staining techniques. Tissue sections were incubated with either Lu-5 mAb for 30 minutes or anti-CEA mAb for 60 minutes. Sections were washed and incubated with gamma-chain specific goat anti-mouse IgG-HRP antibody (16 μg/ml) of for 30 minutes. After washing, sections were stained with DAB chromagen for 5 minutes.

### Quantitative Real Time-PCR

Specific primers for C2GNT1 (Gen Bank Accession number NM_001490) were designed as follows: the forward primer was 5'-GAT GTC ACC TGG AAT CAG CA-3' and the reverse primer was 5'-GCA GCA ACG TCC TCA GCA T-3'. The cDNAs were amplified in an Applied Biosystems 7500 Real-Time PCR system using a reaction volume of 7.5 μl containing 1× SYBR Green PCR Master Mix (Applied Biosystems, Foster City, CA) and 15 pmol/μL of each primer. The PCR conditions included one cycle at 95°C for 10 minutes followed by 50 cycles of denaturation at 95°C for 15 seconds; annealing at 58°C for 23 seconds; and extension at 72°C for 35 seconds. The dissociation was performed in cycles of 95°C for 15 seconds; annealing at 60°C for 60 seconds; and extension at 95°C for 15 seconds. The relative amount of C2GNT1 transcripts in each sample was normalized to the house keeping gene β-actin by removing the cycle threshold (Ct) value of β-actin from Ct value of C2GNT1 (ΔCt). The fold difference was calculated by subtracting the ΔCt of the test sample from that of the control sample to give ΔΔCt, and fold difference = 2-^ΔΔCt ^[45]. Absolute transcript expression values for C2GNT1 and β-actin beyond 45 cycles were considered to be below detectable levels and those data were eliminated. Each assay was done in duplicate and melt curves were conducted for each reaction to guarantee that a single product was amplified. The PCR products were further confirmed by gel electrophoresis.

### Histologic and RT-PCR Grading System for Tissues

The intensity of CHO-131 mAb staining for C2-O-sLe^x ^in immunoreactive tissues was scored using a semiquantitative system from 0+ to 3+. Score 0+, no visible immunostaining; score 1+, mild immunostaining; score 2+, moderate immunostaining; and score 3+, strong immunostaining. A score of 0+ to 1+ indicated low reactivity with CHO-131 mAb and a score of 2+ to 3+ indicated high reactivity. Cells were evaluated in five different high-power field areas for each tissue section. Each tissue specimen was scored independently by two pathologists. Any discordant scores were re-evaluated and scored again on the basis of a consensus opinion.

### Statistical Analysis

All statistical analyses were implemented using SAS statistical software, version 9.1. In all statistical tests, a p-value of ≤ 0.05 was considered significant. For analysis of the expression of C2-O-sLe^x ^in tissue sections of primary colorectal adenocarcinomas and those carcinomas metastatic to the liver, (as indicated by CHO-131 mAb staining), tissues were grouped as either well, moderately, or poorly differentiated. Malignant tissues were also categorized as low expression (score 0+ to 1+) and high expression (score 2+ to 3+) for C2-O-sLe^x^. The Pearson's chi-square test was applied to groups to test any associations between the categorical variables and odds ratios were calculated by the methods of Cochran, Mantel, and Haenszel. The levels of C2GnT1 mRNA in normal colorectal tissues and in well, moderately, and poorly differentiated colorectal adenocarcinomas, but not in liver tissues, was analyzed using the Kruskal-Wallis test with a 5% significance level. The subgroup comparisons between the well, moderate, and poor groups with the normal group were adjusted by the Bonferroni method.

## Results

### C2-O-sLe^x ^was detected in colorectal adenocarcinomas and metastatic liver tumors by immunohistochemistry

**Figure 2:**
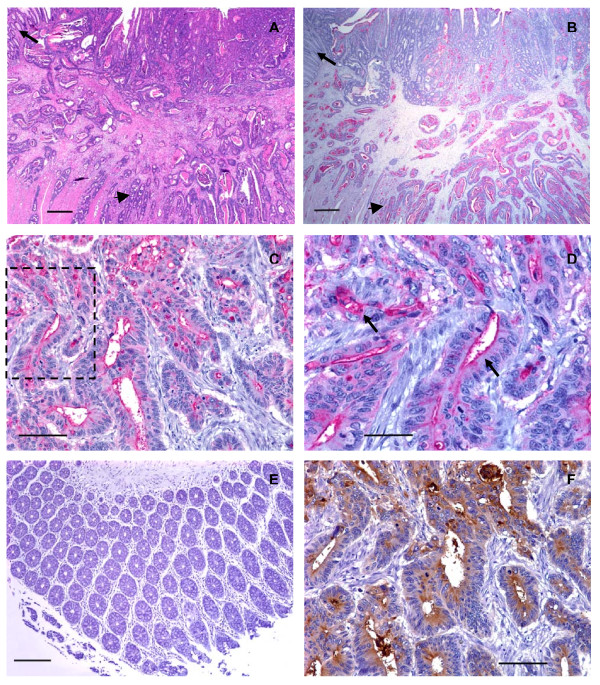
**Human well differentiated colorectal adenocarcinomas expressed low levels of C2-O-sLe^x^**. (A) Photomicrograph of a tissue section of a well differentiated colorectal adenocarcinoma stained with hematoxylin and eosin, scale bar = 500 μm. (B) A serial section of the same tissue as shown in (A) stained with CHO-131 mAb (15 μg/ml). Red color indicates positive reactivity with the CHO-131 mAb, scale bar = 500 μm. In (A) and (B) the arrow indicates adjacent normal colorectal mucosa. The arrowhead indicates nests of neoplastic cells in the tunica submucosa and muscularis. (C) Another well differentiated colorectal adenocarcinoma stained with CHO-131 mAb. Red color indicates positive reactivity with the CHO-131 mAb, scale bar = 100 μm. (D) Increased magnification of the boxed area shown in (C) demonstrating red stained CHO-131^+ ^cells. Arrows in (D) indicate cytoplasmic and luminal reactivity with CHO-131 mAb, scale bar = 50 μm. (E) Mucosa of normal colorectal epithelium stained with CHO-131 mAb. Note the absence of red color, indicating a lack of reactivity with the CHO-131 mAb, scale bar = 150 μm. (F) A serial section of the same tissue as in (C) and (D), stained with CEA mAb (1.6 μg/ml). Brown color indicates positive reactivity with CEA mAb, scale bar = 100 μm. All tissue sections were 4 μm thickness. Figures (A) and (B) 20× magnification, (C) 200× magnification, (D) 400× magnification, (E) 100× magnification, and (F) 200× magnification. Mayer's hematoxylin was used as a counterstain for tissues stained with CHO-131 and CEA mAbs. Representative sections from multiple stained tissues are shown.

**Figure 3:**
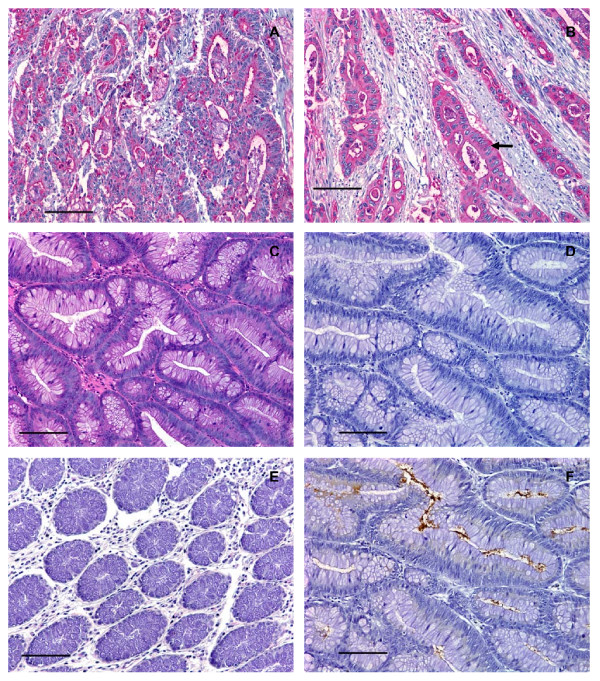
**Human colorectal adenocarcinomas, but not colorectal adenomas, expressed high levels of C2-O-sLe^x^**. (A) Photomicrograph of a tissue section of a moderately differentiated colorectal adenocarcinoma stained with CHO-131 mAb (15 μg/ml). Red color indicates positive cytoplasmic and luminal reactivity with CHO-131 mAb. (B) A poorly differentiated colorectal adenocarcinoma stained with CHO-131 mAb. Notice the red color indicating positive cytoplasmic reactivity of neoplastic cells (arrow). (C) A colorectal adenoma stained with hematoxylin and eosin. (D) A serial section of the same tissue as in (C), stained with CHO-131 mAb. Notice the absence of red color. (E) Mucosa of normal colorectal epithelium stained with CHO-131 mAb. (F) A serial section of the same tissue as in (C) and (D), stained with CEA mAb (1.6 μg/ml). The brown color indicates positive reactivity with CEA within the lumens of glands and in the cellular cytoplasm. All tissue sections were 4 μm thickness; scale bars = 100 μm, 200× magnification. Mayer's hematoxylin was used as a counterstain for tissues stained with CHO-131 and CEA mAbs. Representative sections from multiple stained tissues are shown.

There was a unique pattern of reactivity with CHO-131 mAb in cancer tissues. CHO-131 mAb positively stained both the cytoplasm and luminal surfaces of glandular structures in all colorectal adenocarcinomas. The intramucosal aspects of each adenocarcinoma stained less intensely with the CHO-131 mAb and the staining pattern was irregular. The deeper submucosal and muscularis areas of intestine that were infiltrated by tumor cells demonstrated a more uniform, intense staining for CHO-131 mAb and this finding was especially prominent at the advancing edge of the tumor with the deepest invasive components (Figure 2A, B). The positive reactivity with CHO-131 mAb was most prominent in neoplastic colorectal glands of well to moderately differentiated colorectal adenocarcinomas (Figure 2C, D and Figure 3A).

As colorectal adenocarcinomas progressed from a well to poorly differentiated status, proportionately greater numbers of carcinomas displayed increased areas of solid tumor growth that lacked glandular structures. Eight of 8 poorly differentiated colorectal adenocarcinomas examined demonstrated positive reactivity with CHO-131 mAb. We detected high (2+ to 3+) positive staining in 6 of 8 of these tissues. In two of these 6 positively stained, poorly differentiated adenocarcinomas approximately 95% of the tumor consisted of glandular structures that were highly reactive (3+ intensity) with CHO-131 mAb. An example is shown in Figure 3B. In the remaining 4 poorly differentiated adenocarcinomas, only 5–20% of the neoplastic tissue consisted of glandular structures that were highly reactive with CHO-131 mAb. Instead, the majority of the tumor consisted of non-reactive fibrous tissue. Lastly, we observed low (1+) reactivity with CHO-131 mAb in the remaining 2 of 8 poorly differentiated colorectal adenocarcinomas with minimal glandular structures (less than 10% of the total tumor). Glandular structures in normal colorectal epithelium and in colorectal adenomas did not react with CHO-131 mAb (Figure 2E and Figure 3C, D, E).

In our positive controls, we observed that between 75 – 100% of the total tumor area in all colorectal adenocarcinomas stained positively with the anti-CEA mAb, regardless of differentiation status. In colorectal adenocarcinomas, reactivity with anti-CEA mAb was localized to the luminal and sub-membranous regions of the cytoplasm and the staining pattern mimicked that of CHO-131 mAb staining (Figure 2F). As expected, all colorectal adenomas showed positive reactivity with the anti-CEA mAb in 25 – 50% of the total area of each tissue. A representative colorectal adenoma stained with anti-CEA mAb is shown in Figure 3F. Serial tissue sections of liver metastases of primary colorectal adenocarcinomas that reacted positively with CHO-131 mAb displayed strong positive reactivity with the anti-CEA mAb in similar areas of tissue. In contrast, anti-CEA mAb did not react with normal liver tissues (data not shown). As expected, we observed positive reactivity with the Lu-5 mAb in all normal and malignant colorectal and liver tissues examined (data not shown). In HL-60 cells used as positive controls, the cell membrane and cytoplasm stained strongly (3+ reactivity) with the CHO-131 mAb (data not shown). All tissue specimens stained with isotype matched negative control antibodies were negative for tissue reactivity.

**Figure 4:**
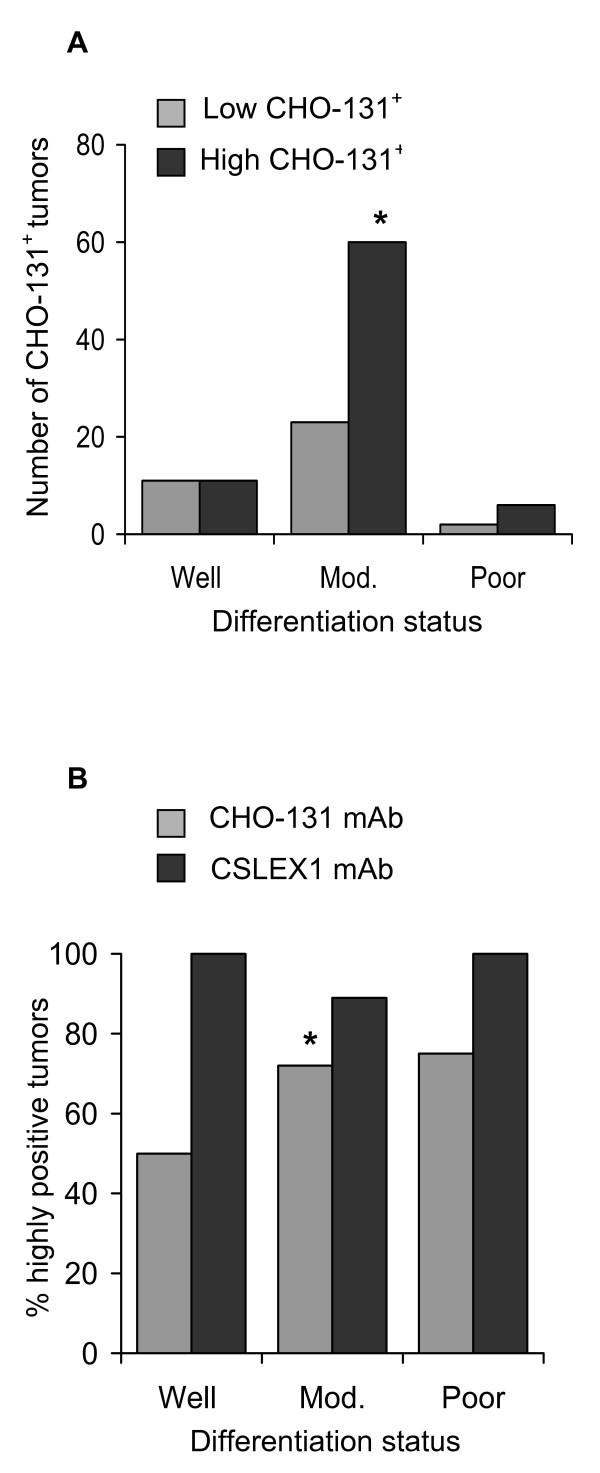
**Moderately differentiated human colorectal adenocarcinomas expressed high levels of C2-O-sLe^x^**. (A) A graph of the number of neoplastic cells in well, moderately, and poorly differentiated colorectal adenocarcinomas that were positive for expression of CHO-131 mAb at low (0+ to 1+ staining intensity) and high (2+ to 3+ staining intensity) levels. (B) A graph of the percentage of colorectal adenocarcinomas that were highly positive for C2-O-sLe^x ^compared to sLe^x^; n = 22, n = 83, and n = 8 for well, moderately, and poorly differentiated colorectal adenocarcinomas stained with CHO-131 mAb that detected C2-O-sLe^x^; n = 11, n = 112, and n = 11 for well, moderately, and poorly differentiated colorectal adenocarcinomas stained with CSLEX1 mAb that detected sLe^x^. *Moderately differentiated colorectal adenocarcinomas expressed significantly higher levels of C2-O-sLe^x ^than well differentiated carcinomas by the Pearson's chi-squared test, p = 0.04.

A summary of the reactivity of colorectal adenocarcinomas with CHO-131 mAb is shown in Table 1. Of the 113 total colorectal adenocarcinomas examined, 22 were well differentiated, 83 were moderately differentiated, and 8 were poorly differentiated. We observed high, (2+ to 3+), positive reactivity with the CHO-131 mAb in 50% (11 of 22) well differentiated, 72% (60 of 83) moderately differentiated, and 75% (6 of 8) poorly differentiated adenocarcinomas. Colorectal adenocarcinomas in the moderately differentiated group displayed significantly higher levels of positive CHO-131 mAb staining compared to those adenocarcinomas in the well differentiated group (Pearson's chi-squared test, p-value = 0.04, odds ratio = 0.38) (Figure 4A). Although the majority of poorly differentiated adenocarcinomas examined reacted positively with CHO-131 mAb at high (2+ to 3+) intensity, significant differences in reactivity were not observed when poorly differentiated colorectal adenocarcinomas were compared with the well and moderately differentiated groups.

**Table 1:** C2-O-sLe^x ^was expressed in human primary colorectal adenocarcinomas.

	CHO-131 mAb staining reactivity in colorectal adenocarcinomas			
	
Differentiation status	0+	1+	2+	3+
Well differentiated	1	10 (5)	5 (4)	6 (5)

Moderately differentiated	13	10 (1)	28 (12)	32 (14)

Poorly differentiated	0	2 (0)	2 (0)	4 (2)

10 benign adenomas were negative for C2-O-sLe^x ^expression by CHO-131 staining. 28 normal tissues were negative for C2-O-sLe^x ^expression by CHO-131 staining. The brackets indicate the number of tissues where greater than 50% of the total tumor mass stained positively with CHO-131 mAb.

Colorectal adenocarcinomas metastatic to the liver but not normal liver tissues were positively reactive with the CHO-131 mAb. CHO-131 mAb staining was detected in the cytoplasm and at the surfaces of glandular lumens of neoplastic cells. Of the 46 well, moderately and poorly differentiated metastatic liver tumors examined, 40 were positively stained with CHO-131 mAb and 35 were strongly positive (2+ to 3+ staining) (Table 2).

**Table 2:** C2-O-sLe^x ^was expressed in colorectal adenocarcinomas metastatic to the liver.

Differentiation status	CHO-131 mAb staining reactivity in metastatic liver tumors			
	
	0+	1+	2+	3+
Well differentiated	0	0	1	2

Moderately differentiated	6	5	10	16

Poorly differentiated	0	0	3	3

5 normal liver tissues did not stain with CHO-131.

**Figure 5:**
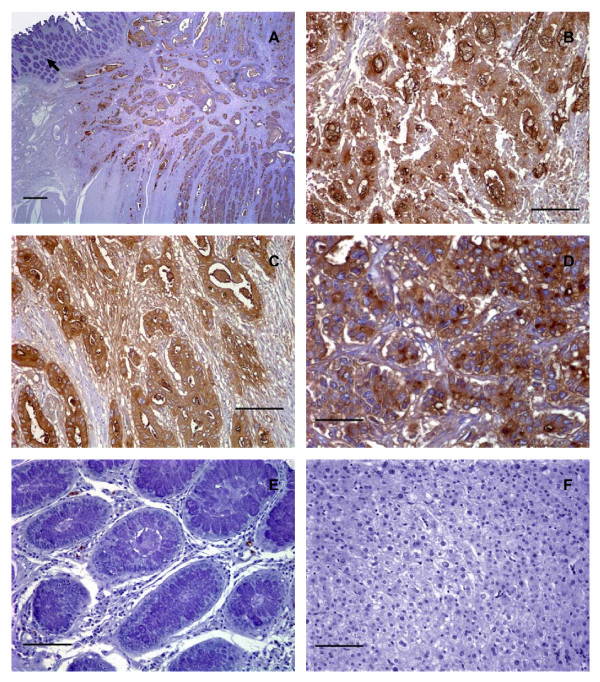
**Human primary colorectal adenocarcinomas and metastatic liver tumors expressed sLe^x^**. (A) Photomicrograph of a serial tissue section of the same well differentiated colorectal adenocarcinoma as shown in Figure 2A stained with CSLEX1 mAb (15 μg/ml). Brown color indicates positive reactivity with the CSLEX1 mAb. The arrow indicates adjacent normal colorectal mucosa that lacks reactivity with CSLEX1 mAb, scale bar = 500 μm. (B) A moderately differentiated colorectal adenocarcinoma stained with CSLEX1 mAb. Brown color indicates positive reactivity with CSLEX1 mAb, scale bar = 100 μm. (C) A serial tissue section of the same poorly differentiated colorectal adenocarcinoma as in Figure 3B stained with CSLEX1 mAb (15 μg/ml). Brown color indicates positive reactivity with CSLEX1 mAb, scale bar = 100 μm. (D) A moderately differentiated metastatic colorectal adenocarcinoma in liver stained with CSLEX1 mAb. The cytoplasm of neoplastic cells reacted positively with CSLEX1 mAb as indicated by the brown color, scale bar = 50 μm. (E) Mucosa of normal colorectal epithelium stained with CSLEX1 mAb. Note the absence of brown color, indicating a lack of reactivity with CSLEX1 mAb, scale bar = 100 μm. (F) Normal liver stained with CSLEX1 mAb. Note the absence of brown color, indicating a lack of reactivity with the CSLEX1 mAb, scale bar = 100 μm. All tissue sections were 4 μm thickness. Figure (A) 20× magnification, (B) and (C) 200× magnification, (D) 400× magnification, (E) and (F) 200× magnification. Mayer's hematoxylin was used as a counterstain. Representative sections from multiple stained tissues are shown.

**Figure 6:**
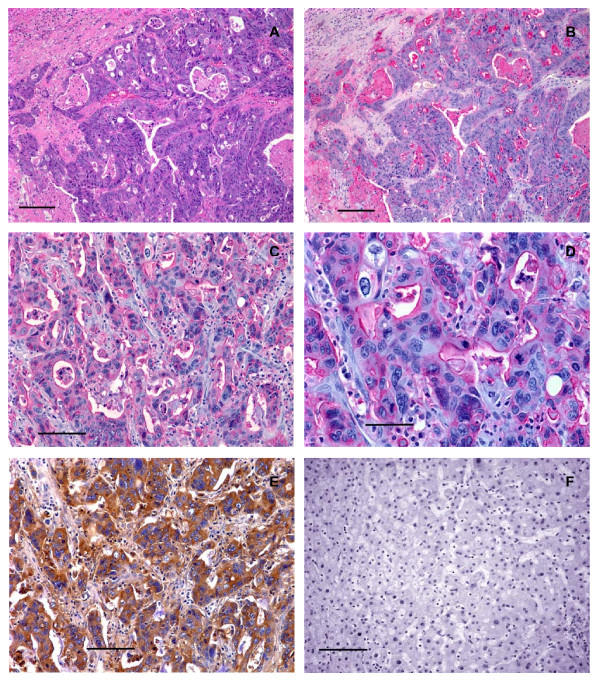
**Human metastatic liver tumors of primary colorectal adenocarcinomas expressed C2-O-sLe^x^**. (A) Photomicrograph of a tissue section of metastatic colorectal adenocarcinoma tumors in liver stained with hematoxylin and eosin, scale bar = 150 μm. (B) A serial section of the same tissue as shown in (A), stained with CHO-131 mAb (15 μg/ml). Red color indicates positive reactivity with the CHO-131 mAb, scale bar = 150 μm. (C) A different tissue section of metastatic colorectal adenocarcinoma in liver stained with CHO-131 mAb, scale bar = 100 μm. (D) A higher magnification of the same tissue section as in (C), stained with CHO-131 mAb (15 μg/ml), scale bar = 50 μm. (E) A serial section of the same tissue as shown in (C) and (D), stained with CEA mAb (1.6 μg/ml) as a positive control. Brown stained cells are positive for CEA mAb, scale bar = 100 μm. (F) Normal liver stained with CHO-131 mAb (15 μg/ml). Note the absence of red color, indicating a lack of reactivity with CHO-131 mAb, scale bar = 100 μm. All tissue sections were 4 μm thickness. Figures (A) and (B) 100× magnification, (C) 200× magnification, (D) 400× magnification, (E) and (F) 200× magnification. Mayer's hematoxylin was used as a counterstain for tissues stained with CHO-131 and CEA mAbs. Representative sections from multiple stained tissues are shown.

We assessed whether the reactivity of CHO-131 mAb and CSLEX1 mAb overlapped. All primary colorectal adenocarcinomas and metastatic liver tumors examined reacted positively with CSLEX1 mAb. The percentage of colorectal adenocarcinomas that were highly reactive with CHO-131 mAb (2+ to 3+ staining intensity) are shown in Figure 4B. The cytoplasm of glandular structures in primary colorectal adenocarcinomas and metastatic tumors was positively stained with CSLEX1 mAb and reactivity was not related to differentiation status of the tumor. We observed strong, highly positive 2+ to 3+ reactivity with CSLEX1 mAb in similar tissue locations of colorectal adenocarcinomas and metastatic liver tumors to those areas that positively reacted with CHO-131 mAb (Figure 5A, B, C, D and Table 3). However, positive staining with CSLEX1 mAb was more widely distributed in tissues than that with CHO-131 mAb. Normal colonic mucosa and hepatocytes in normal liver lacked reactivity with CSLEX1 mAb (Figure 5E, F). Representative sections of metastatic liver tumors that were positively stained with CHO-131 mAb are shown in Figure 6.

**Table 3:** C2-O-sLe^x ^was compared to sLe^x ^expression in primary colorectal adenocarcinomas and metastatic liver tumors.

	Differentiation Status					
	**Well differentiated**		**Moderately differentiated**		**Poorly differentiated**	

**Antibody staining reactivity in colorectal adenocarcinomas**	0+	(NE)	0+	(1+)*	NE	(NE)
	1+	(2+)*	1+	(1+)	1+	(3+)*
	2+	(2+)	2+	(3+)*	2+	(3+)*
	3+	(3+)	3+	(3+)	3+	(3+)

**Antibody staining reactivity in metastatic liver tumors**	NE	(NE)	0+	(0+)	NE	(NE)
	NE	(NE)	1+	(2+)*	NE	(NE)
	2+	(2+)	2+	(2+)	2+	(3+)*
	3+	(2+)	3+	(3+)	3+	(3+)

Serial sections of individual tissues were stained with CHO-131 mAb that detects C2-O-sLe^x ^and CSLEX1 mAb that detects sLe^x^. The numbers in the body of the table indicate the intensity of reactivity of a representative single tissue with CHO-131 mAb. The bracketed numbers indicate the intensity of reactivity of a serial section of the same tissue with CSLEX1 mAb. NE = not examined. * indicates a difference in reactivity between CHO-131 and CSLEX1 mAbs.

### C2GnT1 mRNA levels were upregulated in colorectal adenocarcinomas

**Figure 7:**
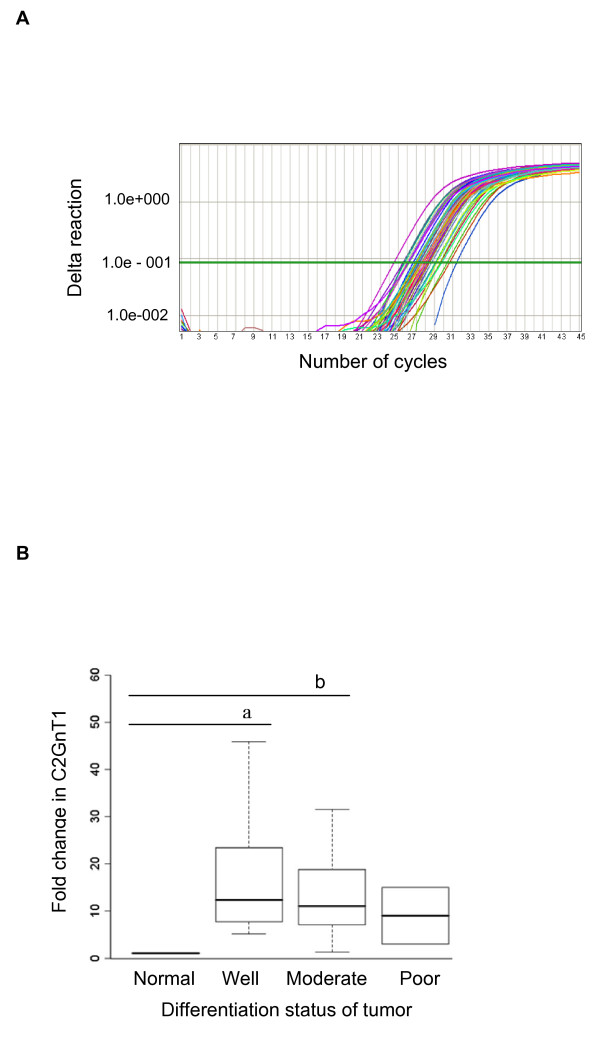
**C2GnT1 mRNA levels were upregulated in human colorectal adenocarcinomas compared to normal tissues**. (A) Graph of amplification plots obtained by the Applied Biosystems 7500 Real-Time PCR system for C2GnT1 mRNA expression. (B) A box-plot showing the results of semi-quantitative RT-PCR analysis. The distribution of mRNA gene expression is shown as a fold change in C2GnT1 mRNA expression in normal colorectal tissues and in well, moderately, and poorly differentiated colorectal adenocarcinomas. A Kruskal-Wallis non-parametric test was performed for statistical comparison among the groups. ^a ^Denotes a significant difference (p = 0.015) between normal and well groups; ^b ^Denotes a significant difference (p = 0.025) between normal and moderate groups.

We used RT-PCR to analyze C2GnT1 mRNA levels in 20 well, 15 moderately, and 2 poorly differentiated colorectal adenocarcinomas and we compared these mRNA expression levels to those in 5 normal colorectal tissues. The amplification plot from the RT-PCR assay is shown in Figure 7A. As shown in Figure 7B, C2GnT1 mRNA levels were more than 15 times higher in the well and moderately differentiated colorectal adenocarcinomas when compared to normal colorectal tissues, and this difference was statistically significant (p = 0.015 and p = 0.025 respectively using the Kruskal-Wallis test). Although C2GnT1 mRNA levels were more than 9-fold upregulated in poorly differentiated colorectal adenocarcinomas compared to normal colorectal tissues, the change was not significant.

## Discussion

The C2-O-sLe^x ^motif on leukocytes confers much higher affinity binding to the selectins than sLe^x ^[19,20]. Detection of C2GnT1 mRNA transcripts, and in some instances sLe^x^, have been reported to positively correlate with the invasiveness and metastasis of human colorectal, pulmonary, and oral cavity carcinomas [35-37]. These studies indirectly imply that C2-O-sLe^x ^expression may be increased on carcinoma cells. However, the presence of the sLe^x ^epitope on cells is not always indicative of C2GnT1 gene expression because sLe^x ^can decorate other glycoconjugates such as glycoproteins, glycolipids, N-glycans and O-glycans [46-51], that may have different functions from those of C2-O-sLe^x^. The direct detection and evaluation of C2-O-sLe^x ^in the progression and metastasis of human colorectal adenocarcinomas has not been performed. We hypothesized that C2-O-sLe^x ^– a cell surface expressed high affinity selectin ligand – is expressed on carcinoma cells and is associated with malignant progression and metastasis. Our hypothesis was based on evidence suggesting that similar adhesion mechanisms regulate extravasation of leukocytes and carcinoma cells from the circulatory system into tissues [21-24].

The major findings of our study were that C2-O-sLe^x^, a glycan detected by the CHO-131 mAb, was abundantly present in human colorectal adenocarcinomas and metastatic liver tumors but was absent in colorectal adenomas and normal colorectal and liver tissues. As colorectal adenocarcinomas progressed from well to moderately differentiated status, significantly greater numbers of the tumors examined displayed high levels of C2-O-sLe^x ^on the cell surface where more than 50% of the total tumor mass was positive for C2-O-sLe^x ^expression. Furthermore, we detected a significant increase (greater than 15-fold) in average C2GnT1 mRNA levels in well and moderately differentiated colorectal adenocarcinomas compared to normal colorectal tissues by RT-PCR. Our findings are novel because, to our knowledge, we are the first to provide direct evidence that C2-O-sLe^x ^on colorectal adenocarcinomas is a tumor antigen and that increased expression of C2-O-sLe^x ^is associated with more advanced disease.

We detected high expression of C2-O-sLe^x ^in the majority (6 of 8) of poorly differentiated adenocarcinomas. We observed the highest C2-O-sLe^x^expression (3+ staining intensity) in 2 of 6 malignant tissues where over 95% of the tumor mass consisted of neoplastic glandular structures. We also found a 9-fold increase in C2GnT1 mRNA levels in poorly differentiated adenocarcinomas compared to normal colorectal tissues that was not statistically significant. These results were surprising because we expected poorly differentiated adenocarcinomas to have the highest expression of cell surface C2-O-sLe^x ^and corresponding upregulation of C2GnT1 mRNA. Poorly differentiated colorectal adenocarcinomas account for 5–10% of all cases of colorectal cancer and are regarded as highly malignant. These tumors can be further classified into solid type and non-solid type patterns of growth where the solid type pattern is predominantly non-glandular. We observed that C2-O-sLe^x ^expression is mainly localized to neoplastic glands. In the majority (6 of 8) of the poorly differentiated colorectal adenocarcinomas that we examined there was minimal glandular differentiation compared with well and moderately differentiated adenocarcinomas. This finding is a likely explanation for the decrease in C2GnT1 mRNA expression and reactivity with CHO-131 mAb that we observed in poorly differentiated tumors. Because solid type poorly differentiated colorectal adenocarcinomas are associated with a better prognosis compared to the non-solid type [52], we speculate that the intense reactivity with CHO-131 mAb that we observed in highly glandular non-solid type poorly differentiated colorectal adenocarcinomas is an indicator of a poor prognosis. This possibility remains to be investigated in the future using a larger sample size of this subgroup of tissues. Another possible reason for the decrease in C2GnT1 mRNA levels that we observed in poorly differentiated colorectal adenocarcinomas is that the tissues used for RT-PCR and those used for immunohistochemistry were from different sources and we could not make a direct correlation between C2GnT1 mRNA levels and C2-O-sLe^x ^levels in the same patient. The small sample size obtained for the poorly differentiated adenocarcinomas used in the immunohistochemical staining (n = 8) and RT-PCR analysis (n = 2) may be an additional explanation for this discrepancy.

Intestinal mucins are glycoproteins characterized by dense O-glycosylation on serine and threonine amino acids. Colonic mucins have four main O-glycan core structures (1–4). Of these structures, core 2 beta 1,6 branching is critical for biosynthesis of sLe^x ^on O-glycans. Core 2 branching can be synthesized by at least three enzymes in the C2GnT family, C2GnT1 or C2Gnt-L (leukocyte-type) [53], C2GnT2 or C2GnT-M (mucin-type) [54] and C2GnT3 or C2GnT-T (thymus-associated) [54,55]. C2GnT-M can form multiple branched structures: core 2 type branching, core 4 type branching, and blood group antigen I. C2GnT-M is highly expressed in healthy colonic mucosa and is replaced by C2GnT-L in colon cancer tissues [56]. Recently, Huang et al. reported that C2GnT-M is downregulated in colorectal cancer and suppresses colon cancer cell growth [57]. In this study the differentiation status of the tumors was not examined. C2GnT-L and C2GnT-M may have distinct but overlapping functions in the progression of colorectal carcinomas. We observed a considerable increase in C2CnT-L expression at the protein and mRNA levels as colorectal carcinomas progressed from well to moderately differentiated. We speculate that in the early stages of colorectal cancer carcinogenesis, C2GnT-M may predominate in colonic tissues with developing malignancy and act as a control mechanism to inhibit the growth of colon cancer cells. As the tumor progresses to more advanced stages, upregulated C2GnT-L activity may predominate and be associated with a corresponding increase in C2-O-sLe^x ^expression on colorectal cancer cells. The biochemical pathway of C2-O-sLe^x ^synthesis is complex involving several sequential glycosyltransferase reactions. We cannot rule out that other glycosyltransferases in addition to C2GnT1 may also be upregulated in colorectal adenocarcinomas resulting in the increased C2-O-sLe^x ^levels that we observed in our study. Nevertheless, our findings indicate that high levels of C2-O-sLe^x^, detected by the CHO-131 mAb, corresponded to measurable upregulation of the C2GnT1 gene as colorectal adenocarcinomas progressed in malignancy.

C2-O-sLe^x ^is also present on most leukocytes but this cell type was easily morphologically distinguishable from carcinoma cells by hematoxylin and eosin staining. Furthermore, we performed immunohistochemical staining with the Lu-5 mAb that detects epithelial cells to verify that C2-O-sLe^x ^positive malignant cells were epithelial in origin. Thus, we found that the C2-O-sLe^x ^antigen on colorectal adenocarcinomas was associated with a more advanced tumor. Similarly to leukocytes, C2-O-sLe^x^may represent a high affinity glycan ligand for the selectins.

Providing direct evidence for the expression of C2-O-sLe^x ^on carcinoma cells is important because this oligosaccharide serves as a high affinity ligand for the selectins during leukocyte migration into sites of inflammation and secondary lymphoid organs. Similarly, C2-O-sLe^x ^may function as a high affinity selectin ligand on carcinoma cells that promotes adhesion of malignant cells to endothelium and subsequent metastasis. The direct demonstration of C2-O-sLe^x ^on the cell surface is essential because other enzymes such as the glycosyltransferase ST3 beta-galactoside alpha-2,3-sialyltransferase 1 can compete with C2GnT1 in carcinoma cells and inhibit the conversion of core 1 O-glycans to core 2 [38].

It is well established that levels of sLe^x ^increase during progression of colorectal adenocarcinomas from nonmetastatic to metastatic tumors and there is an inverse correlation with postoperative survival [10]. However, a direct investigation of the expression of C2-O-sLe^x ^specifically at various stages of malignant colorectal tumor growth and in normal colorectal tissues had not been performed previously. Similarly to leukocytes, expression of C2-O-sLe^x ^may promote much greater selectin-mediated adhesiveness of cancer cells to endothelium than sLe^x ^on other structures resulting in enhanced metastatic ability. Indeed, we have shown that in two colorectal adenocarcinoma cell lines, the cell line expressing C2-O-sLe^x^, but not the cell line lacking C2-O-sLe^x^, bound avidly to E-selectin under shear stress conditions simulating the vasculature [43]. Importantly, sLe^x ^can terminally decorate other cell surface macromolecules that may not be high affinity selectin ligands including proteins containing N-glycans, non-core 2 O-glycans, and glycolipids. Recently, CHO-131 mAb was found to stain a subset of cutaneous lymphocyte-associated antigen (CLA) T cells. P-selectin binding activity was significantly greater for CHO-131^+ ^CLA^+ ^T cells than for CHO-131^- ^CLA^+ ^T cells. C2-O-sLe^x ^expression in CLA^+ ^T cells appears to differentially regulate high affinity binding to P-selectin [58]. In colorectal adenocarcinomas, differences in C2-O-sLe^x ^expression may similarly regulate adhesion to the selectins and metastasis.

Sialylated Lewis structures such as sLe^x ^and sLe^a ^are highly expressed on melanomas and various types of carcinomas including colon, gastric, pancreatic, squamous cell, and breast and expression levels correlate with an advanced stage of disease and poor patient prognosis [2-7,10,59-62]. Moreover, sLe^x ^is a selectin ligand capable of mediating the binding of tumor cells to endothelia, platelets, and neutrophils [25,28]. It is intriguing to speculate that C2-O-sLe^x ^in some instances is the moiety mediating these reported interactions. In particular, C2-O-sLe^x ^has recently been shown to be expressed on cutaneous T cell lymphomas and is a ligand for P- and E-selectin [63]. We report here that in colorectal adenocarcinomas and metastatic liver tumors, C2-O-sLe^x ^expression was present in similar regions of tissue as that noted for sLe^x ^expression but the latter was more widely distributed. This result was expected because CSLEX1 mAb recognizes the sLe^x ^epitope alone that can be present on several types of glycan structures including core 2 O-glycans [64]. In contrast, CHO-131 mAb specifically detects sLe^x ^on core 2 O-glycans which represents a unique subset of glycoproteins present on tumor cells.

The development of metastasis is an important factor in determining the outcome of patients with colorectal adenocarcinomas. Metastatic involvement of lymph nodes or metastases into distant organs greatly decreases the median patient survival [65]. The liver is the primary site of metastasis for colorectal adenocarcinomas [66]. Interestingly, sLe^x ^expression by carcinomas has been proposed to enhance the ability of tumor cells to metastasize to the liver through E-selectin mediated interactions [3,27,32,67] and sLe^x ^epitopes are overexpressed in liver metastases of colorectal adenocarcinomas [3,8,68]. In our study, all normal liver tissues lacked reactivity with the CHO-131 mAb. We found that 87% (40 of 46) of liver metastases of primary colorectal adenocarcinomas examined stained positively with the CHO-131 mAb. A subset (35 of 40) of these positive tissues exhibited high (2+ to 3+) staining intensity with CHO-131 mAb indicating that C2-O-sLe^x ^was abundantly present on metastatic liver tumors. Of note is that liver metastases of primary colorectal adenocarcinomas but not normal liver tissues expressed C2-O-sLe^x^. Our findings suggest that high levels of the C2-O-sLe^x ^antigen was preserved on the surface of colorectal adenocarcinomas cells as they migrated from the primary tumor to the liver, a common site of metastasis. We speculate that C2-O-sLe^x ^expression may be specifically upregulated during metastasis to promote the metastatic ability of colorectal adenocarcinomas.

In summary, our results demonstrate that C2-O-sLe^x ^is highly expressed in colorectal adenocarcinomas and liver metastases but not on benign colorectal and liver tissues. Our observations suggest that upregulation of C2GnT1 gene expression is correlated with increased cell surface expression of C2-O-sLe^x^. C2-O-sLe^x ^is an important high-affinity glycan ligand for the selectins on leukocytes and cutaneous T cell lymphomas [16,17,19,20,63]. Additional research is needed to determine whether C2-O-sLe^x ^is a higher-affinity selectin binding ligand than sLe^x ^and its function in the progression and metastasis of colorectal adenocarcinomas.

## Conclusion

Our observations indicate that C2-O-sLe^x ^is a tumor-associated antigen in colorectal adenocarcinomas and metastatic liver tumors. Furthermore, C2-O-sLe^x ^expression may represent an early predictor of progression and metastasis of colorectal adenocarcinomas. C2GnT1 is a candidate as a novel target for regulation of C2-O-sLe^x ^synthesis and is potentially important therapeutically to control carcinoma dissemination. We are currently investigating the role of carcinoma associated C2-O-sLe^x ^compared to sLe^x ^in adhesion to the selectins, tumor invasion, and metastasis. We are also examining the usefulness of C2-O-sLe^x ^as a practical prognostic tool to predict the long-term survival of cancer patients.

## Competing interests

The authors declare that they have no competing interests.

## Authors' contributions

CAS designed the study and coordinated its implementation, carried out the immunohistochemical studies, analyzed and interpreted all data, and drafted and revised the manuscript for important intellectual content; MF carried out the RT-PCR studies, helped to draft the manuscript, and was involved in critical revision of the manuscript; GM participated in the immunohistochemical studies, and was involved in revising the manuscript critically; HEG carried out histopathological analysis of the tissue samples from colon and liver and was involved in revising the manuscript critically; JJ carried out histopathological analysis of the tissue samples from colon and liver and was involved in the study design; QC provided the statistical analysis of the data and assisted in the revision of the manuscript; BW provided the CHO-131 mAb, and critically revised the manuscript for important intellectual content. All authors read and approved the final submitted version of the manuscript.

## Pre-publication history

The pre-publication history for this paper can be accessed here:

http://www.biomedcentral.com/1471-2407/9/79/prepub
